# Accelerated Aging of Tapes Applied to Secure Criminal Contact Traces—Effect on Physio-Mechanical and Safety Behavior

**DOI:** 10.3390/ma18092012

**Published:** 2025-04-29

**Authors:** Magdalena Olejnik, Agnieszka Gutowska, Magdalena Cichecka, Marcin H. Struszczyk, Paweł Kubiak

**Affiliations:** Institute of Security Technologies “MORATEX”, 3 M. Sklodowskiej-Curie Str., 90-505 Lodz, Poland; agutowska@moratex.eu (A.G.); mcichecka@moratex.eu (M.C.); mstruszczyk@moratex.eu (M.H.S.); pkubiak@moratex.eu (P.K.)

**Keywords:** criminal traces, adhesive tapes, accelerated aging, physico-mechanical properties of criminal tapes, criminal tape chemical resistance

## Abstract

Traces of potential contact from a perpetrator for evidence are one of the most frequently secured groups of evidence during the examination of the crime scene and during the examination of material in forensic laboratories. By far the most common way to secure the above-mentioned traces is the use of swabs. The literature reports indicate promising results from the use of adhesive materials for securing contact marks. The products currently on the market are not dedicated to forensic genetics or cause problems with the recovery of protected DNA at the stage of DNA isolation in the laboratory. The aim of this study was to determine the effect of conditions from an accelerated aging process carried out under simulated laboratory conditions (with aging factors as follows: UV radiation, temperature, and humidity level) on the physico-mechanical properties and chemical resistance of adhesive films made of polyethylene (PE) and polypropylene (PP). As part of the research, the influence of storage conditions on the physico-mechanical properties and chemical resistance of developed foil materials used to secure forensic traces was developed and verified. The research was carried out in conditions similar to the real ones, conducting tests of accelerated aging with the following factors: temperature, humidity, and UV radiation.

## 1. Introduction

The collection of trace evidence in criminal investigations, along with its subsequent forensic investigation, serves as a crucial method for the reconstruction of incidents and the identification of the involved parties. This approach is deeply rooted in both historical and epistemological reasons, making it a fundamental aspect of criminal investigation and legal proceedings [1]. Trace examinations are often characterized by a series of detailed microscopical and instrumental analyses, with the objective of fully characterizing the varied features of trace materials and evaluating the significance of the observed properties within the relevant context. Such analyses range from a physical comparison of items to determine if there is a physical fit (i.e., fracture match) to an analysis of the color, construction, and composition of the materials to determine if differences can exclude a potential source [2].

The preservation of evidence in a variety of investigative contexts is frequently associated with trace analysis, encompassing the analysis of fingerprints and blood groups. In instances where conventional methods prove ineffective, trace elements, such as copper, zinc, fluorine, and analogous substances, which are present in organisms in negligible quantities, may offer a valuable and effective investigative tool [3].

While these technologies have the potential to expedite the identification of suspects, they may also reinforce confirmation bias, particularly in instances where an inaccurate scenario directs the investigation from the outset. An experimental study [4] involved 40 experienced Crime Scene Investigators (CSIs) who investigated a mock crime scene to study the influence of rapid identification technologies on the investigation.

It is important to note that not all biological evidence is necessarily visible on first inspection. However, it can be subjected to DNA analysis and is commonly used in criminal proceedings to link an individual to a crime scene [5]. In the context of criminal investigations, the presence of biological forensic traces at crime scenes is crucially important. The origin of these traces, whether from the suspect, the victim, or bystanders, is a crucial element in the investigation process [6]. It is also important to note that an offense may take place in both indoor and outdoor locations. The traces left at the crime scene may not be immediately detectable, but may instead be discovered several hours, days, or even months later [7].

The process of collecting, analyzing, and securing criminal trace evidence has undergone significant changes in recent years. These changes in the methods employed are not only a result of advancements in technology but are also influenced by the various approaches that have been adopted. In 2015, D.A. Stoney et al. [8] based on an analysis of historical approaches, proposed guidelines and outcomes for modern trace evidence methods. Another known method for tracing evidence like fingermarks detection is called MMD (multi-metal deposition) [9]; however, it is not commonly used when compared to others, like Touch DNA, which consists of invisible biological traces deposited through a person’s skin contact with an object or another person. According to [10], a wide range of factors influence the transfer of touch DNA, including the physicochemical characteristics of the surface of the “destination” substrate.

A variety of polymeric techniques and materials are commonly used in securing forensic traces, specified below:

- Security film: Used to secure traces such as fingerprints. These films can be transparent or frosted, allowing traces to be easily viewed without damaging them.
- Polymeric adhesives: Used to collect and hold traces such as fingerprints. These adhesives can be formulated in a variety of ways to ensure effective adhesion to the surfaces where the marks are found.
- Silicones: Used to create footprints, especially for 3D footprints such as shoe prints. Silicone impression compounds allow for the faithful reproduction of details.
- Sprays and powders for making fingerprints visible: they often contain polymers to make the prints more visible on different surfaces. Powders can have different colors and properties, making them easier to use on different types of materials.
- Materials for securing DNA traces: molecularly imprinted polymers (MIPs), fitted to the target specified types of molecules. Those polymers are highly suitable for forensic analysis as they bind to target molecules via non-covalent or covalent interactions in the presence of selected chemicals [11], for example, propylene glycol (PG) [12] and silk [13] have DNA preservative abilities.
- Adhesive tapes: Used to collect traces such as hair or fine particles. These tapes often have special properties to avoid damaging the samples being collected.
- Waxes and impression compounds: Used to preserve traces under difficult conditions, for example, when impressions are made from irregularly textured surfaces.

The following article will discuss the properties of selected low-adhesive films (positive films, PVC-based films, and gelatine-based films) used for criminal trace evidence collection. Typically, forensic-criminal films consist of the following parts:

- A substrate;
- A gelatine-receiving layer;
- A covering film.

Depending on the type of substrate used, to which the gelatin layer is applied, the following examples of films are produced:

- Classic films in different colors and transparency;
- Films on a rubber backing, suitable for recording traces using fingerprint powders;
- Dactyloscopy Datasets: consisting of a transparent film with a classic 0.5–0.6 mm gelatine layer, to which is attached a sheet of suitably adapted paper substrate. The trace will be archived in a mirror [14].

Degradation is a process of structural changes that may be the result of physical or chemical transformations occurring in polymer materials under the influence of long-term exposure to abiotic factors, such as temperature, light, atomic, molecular, and singlet oxygen, radiation y or X, UV radiation, ultrasounds, or chemical substances, including water and steam, as well as mechanical stresses, especially cyclically changing dynamic stresses, as well as under the influence of biological agents involving the activity of microorganisms (biodegradation) [15,16,17,18,19].

The rate of polymer decomposition during the degradation process depends primarily on its physical and chemical properties. Polymers with a simple structure degrade faster than branched ones. The higher the molecular weight of the polymer, the slower the decomposition [16].

A parameter that clearly indicates the degradation of polymer materials is their molecular weight. Its decrease is observed from the beginning of the process and is associated with the shortening of the main polymer chains [15,17,19]. It should be noted, however, that, in some cases, in the first phase of degradation, the degrading agent improves certain properties of the material, especially mechanical strength. This is performed by additional cross-linking in the structure of the material under the influence of, for example, heat. Only in the later phase do other processes occur, such as excessive cross-linking or reduction in molecular weight, which causes the studied properties to deteriorate [15].

Subsequently, a decrease in the strength of the materials is observed, which is an obvious consequence of the rupture of polymer chains, changes in the crystallinity of the polymer, and the polymer structure becoming “loosened” [20].

Degradation also depends on the presence of chemical groups in the molecule that determine the rate of polymer decomposition. These include easily hydrolyzed ester, amide, and urea groups [16].

Most often, changes in the structure of polymers during production are determined by temperature and atmospheric oxygen. Particular attention should be paid to the processing temperature of a given polymer in terms of its degradation temperature, because for some plastics, these temperatures are very similar, which is a significant technological problem [21].

The gradual and civilizational inevitable increase in demand for polymer plastics means that the scientific and research work carried out for years is aimed at explaining the complex mechanisms of the aging process, determining its impact on properties—mainly physical and chemical—as well as assessing the impact of processing conditions on the course of this phenomenon [20].

It is indicated that gamma irradiation is an important factor affecting changes in the structure of polymers. Indeed, it has been shown that exposure of EPDM (ethylene-propylene-diene monomer) elastomer to gamma radiation leads to changes in the crystalline structure of the polymer under study and the rate of crystallization can depend on the radiation dose (intensity) and flow rate [22,23].

Simulation of natural conditions in laboratory apparatus made it possible to intensify the factors affecting the polymer and accelerate the aging process [24,25].

The paper scientifically documents the thesis that the raw material composition of adhesive films and the aging factor used to predict changes occurring during the actual storage of a special product are important in the context of the observed changes in physico-mechanical properties and affect the chemical resistance and forensic functionality of the designed product.

## 2. Materials and Methods

### 2.1. Materials

Table 1 contains the main characteristics of the adhesion tapes used for designing the tape used to protect forensic traces.

### 2.2. Methods

#### 2.2.1. Accelerated Aging Study

The accelerated aging process of the polymeric adhesion tapes was carried out in accordance with the guidelines of ASTM F1980-16 [26] for two case studies: using only temperature, or temperature and humidity as aging factors.

The accelerated aging with temperature was performed in detail, as described in [27] using the following conditions:

- Four storage periods were simulated, i.e., 6 months, 12 months, 18 months, and 24 months, which corresponded to the following incubation times of the tested samples: 18 days, 37 days, 55.5 days, and 74 days;
- At an accelerated aging temperature (T_AA_) of 55 °C;
- Q_10_ = 2.0, where Q is a constant applied to the accelerated aging describing how 10 °C alterations influence the rate of aging;
- Airflow of ≤1 m/s and an atmospheric pressure of 860–1060 hPa [8,9].

The films were placed in a Jouan climatic chamber (Jouan, France), which can obtain a temperature range of 20 to 250 °C (±2 °C), at the specified temperature and time interval listed below.

The accelerated aging using temperature and humidity as aging factors was performed with the following conditions:

- In four storage periods at real/accelerated conditions as follows: 6/18.5, 12/37, 18/55.5, or 24/74 months/days;
- At a relative humidity of 55%;
- At an accelerated aging temperature (T_AA_) of 55 °C;
- Q_10_ = 2.0, where Q is a constant applied to the accelerated aging describing how 10 °C alterations influence the rate of aging;
- Airflow of ≤1 m/s and an atmospheric pressure of 860–1060 hPa [8,9].

#### 2.2.2. Accelerated Aging Using Temperature or Temperature and Humidity as Aging Factors

The films in laboratory conditions were placed in a Binder KBF 240 climatic chamber (Binder GmbH, Germany), which can obtain a temperature range of (20–80) °C ± 2 °C and relative humidity of (50–70)% ± 5% at a set temperature and specified time interval.

#### 2.2.3. Accelerated Aging Study with UV as an Aging Factor

The equivalent of natural radiation energy in one month, in the climatic zone of Central Europe, in a UV aging chamber is obtained within 79 h (the average annual sum of the radiation dose for Poland is 3600 MJ/m^2^) at an intensity of 600 W/m^2^, at the wavelength range of (300–800) nm.

In accordance with the assumptions concerning the accelerated aging process of the tested tapes, two storage periods were simulated: 3 months at real conditions (related to 30 days at accelerated conditions) or 6 months at real conditions (related to 60 days at accelerated conditions) of UV radiation exposure with the use of 42 W/m^2^ radiation intensity [28,29]. The test was carried out at a temperature of 35 °C and a relative humidity of 40%.

The films in laboratory conditions were placed in an aging chamber with a water-cooled xenon arc lamp Alpha+ (Atlas MTT GmbH, Linsengericht, Germany/Mount Prospect, IL, USA) enabling the following conditions to be obtained: wavelength λ = 300–400 nm, irradiance 42 W/m^2^, temperature 35 °C (black bulb temperature 50 °C), and relative humidity 40%; for the specified time interval, the exposure time was of 30 and 60 days.

#### 2.2.4. Determination of the Physico-Mechanical Properties of the Tested Tapes

(A) Areal Density

The areal density of the tested tapes before and after the accelerated aging process was determined according to the PN-ISO 4592:1998 standard [30].

Specimens with an area of (100 ± 0.5) cm^2^ were cut out from the tested material and then weighed on an analytical balance with an accuracy of 0.0001 g. The average areal density was obtained by calculation by dividing the individual specimen masses by their area; the result was expressed in [g/m^2^]. The number of specimens tested was five.

(B) Thickness

The thickness of the tested tapes before and after the accelerated aging process was determined according to the PN-ISO 4591:1999 standard [31].

Rounded specimens with an area of approximately 100 cm^2^ were cut from the tested material, and the thickness was measured to an accuracy of 0.01 mm using a measuring burden of (0.5–1.0) N and a measuring foot of a diameter of (2.5–10.0) mm. The result was expressed in [mm], with an accuracy of 0.01 mm. The number of specimens tested was five.

(C) Density

Samples with measured thicknesses in [mm] and an area of approximately (100.0 ± 0.5) cm^2^ were weighed on an analytical balance.

The density in [g/cm^3^] was determined by a calculation method according to the formula ρ = m/V, where (m) is the mass of the sample and (V) its volume.

(D) Tensile Strength

The tensile strengths of the tested tapes before and after the accelerated aging process were determined according to the PN-EN ISO 527-1:2012 and PN-EN ISO 527-3:2019-01 standards [32,33]. The adhesion tapes were tested after removing the protection cover in two directions (along—direction 1 and across the tape—direction 2).

The test conditions are presented below:

- Type of tensile testing machine: CRE;
- LE: 100 mm;
- Preload: 0.1 MPa;
- Specimen shape type: 2;
- Specimen width: 10 mm;
- Crosshead speed: 200 mm/min;
- Number of specimens: 5 in each direction.

(E) Peel Adhesion

The peel adhesion of the tested tapes before and after the accelerated aging process of temperature and temperature and humidity was determined according to the PN-EN 1939:2007 standard [34]. The research for the UV factor was not presented due to the special size of samples required for the UV accelerated aging process.

The test conditions are presented below:

- Type of tensile testing machine: CRE;
- LE: 100 mm;
- Peel angle: 180°;
- Specimen width: 25 mm;
- Crosshead speed: 300 mm/min;
- Peel direction: in length;
- Plate type: aluminum;
- Number of specimens: 5 in length;
- Roller pressure: 2 kg/cm.

(F) Chemical Resistance Test

Chemical resistance testing involves exposing samples to various chemical reagents and observing whether the material degrades, changes color, or is otherwise damaged.

A chemical resistance test is carried out by placing a measuring cylinder, approximately 4 cm in diameter, on the test surface and then pouring 32 selected chemicals onto it. After 5 min, the chemical effect on the surface test was checked.

Reagents of analytical chemical purity were used for the tests as follows:

- 40% sodium hydroxide (NaOH, Chempur, Piekary Śląskie, Poland);
- 32% hydrochloric acid (HCl, Chempur, Poland);
- 65% nitric acid (HNO_3_. Chempur, Poland);
- Gasoline (Chempur, Poland);
- Acetone (Chempur, Poland).

A sample of the tape was exposed to each of the above-mentioned chemicals for 5 min at room temperature, and then, the resistance of the tape to its action was assessed. The result was defined as positive if there were no changes on the tested surface.

## 3. Results and Discussion

**Figure 1:**
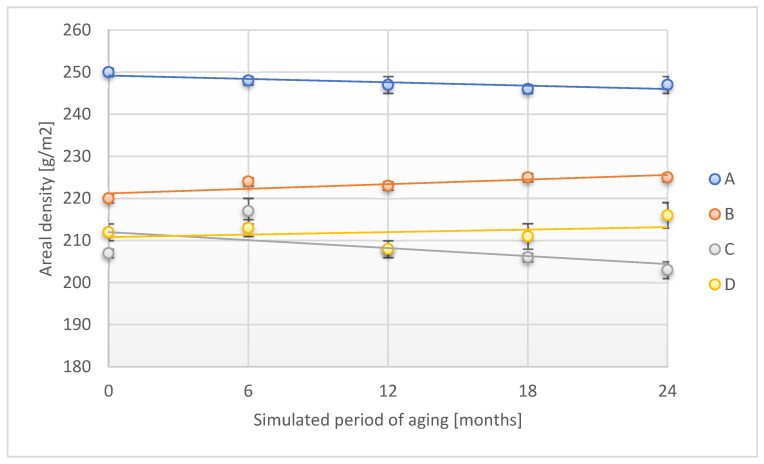
Change in areal density of the tested adhesion tapes during the accelerated aging using temperature: A—POLI TACK 850; B—POLI TACK 854; C—ORAGUARD 210; D—ORAGUARD 215.

Changes in the physico-mechanical properties of tested adhesive tapes designed to protect forensic traces resulting from the accelerated aging process with the use of temperature as an aging factor were evaluated in terms of areal density, density, thickness, peel adhesion, and tensile strength in two directions: longitudinal (direction 1) and transverse (direction 2). Figure 1 shows the changes in the areal density of the various adhesion tapes as an effect of accelerated aging using temperature.

The accelerated aging process corresponding to 24 months of storage under real conditions led to a slight reduction in an areal density of samples: A (by 1.2% compared to the initial sample) and C (by 1.9%) of adhesive tapes designed to protect forensic traces. In the case of samples B and D, an increase in the discussed parameter was observed after the maximum test time by 2.3% and 1.9%, respectively.

**Figure 2:**
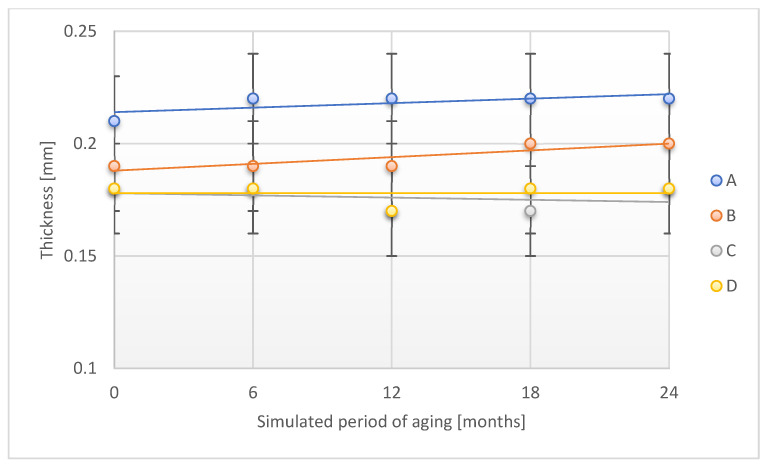
Change in thickness of the tested adhesion tapes during the accelerated aging using temperature: A—POLI TACK 850; B—POLI TACK 854; C—ORAGUARD 210; D—ORAGUARD 215.

In the case of changes in the thickness of the tested adhesive tapes in the accelerated aging process, a slight increase in this parameter was found for A and B samples in the accelerated aging process (Figure 2). This may be related to the effect of increasing temperature on the adhesive layer, which was slightly swollen. C and D samples were characterized by the stability of the discussed parameter during the entire aging process under accelerated conditions.

**Figure 3:**
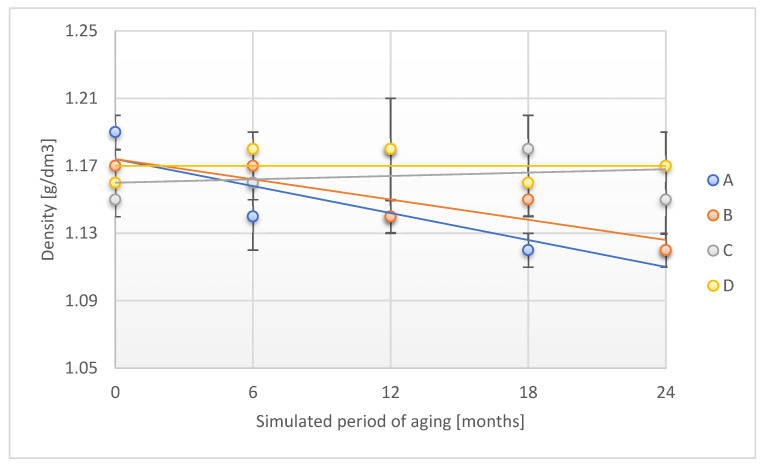
Change in density of the tested adhesion tapes during the accelerated aging using temperature: A—POLI TACK 850; B—POLI TACK 854; C—ORAGUARD 210; D—ORAGUARD 215.

Figure 3 presents the alteration in density of the tested adhesion tapes during the accelerated aging.

A and B samples decreased their density by 5.9% and 4.3%, respectively, after a time corresponding to 24 months of storage under real conditions. The above phenomenon confirms the changes observed when verifying the thickness of the tested adhesion tapes. The density of the C and D samples maintained density stability throughout the aging period.

**Figure 4:**
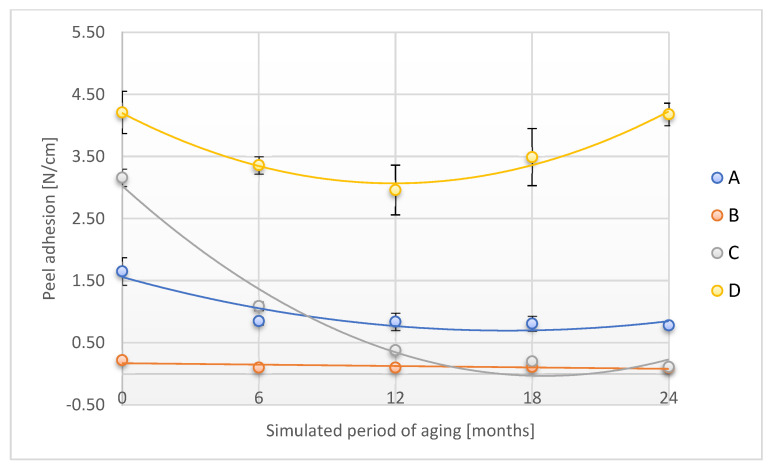
Change in peel adhesion of the tested adhesion tapes during the accelerated aging using temperature: A—POLI TACK 850; B—POLI TACK 854; C—ORAGUARD 210; D—ORAGUARD 215.

The alteration in the peel adhesion of the tested adhesion tapes during the accelerated aging is shown in Figure 4.

Peel adhesion significantly decreased for A, B, and C samples over a study period corresponding to 6 months (samples A and B) and 12 months (sample C) of aging related to real conditions. After the maximum time of accelerated aging tests corresponding to 24 months of storage in real conditions, the described parameter was reduced from 52.7% (A sample) to as much as 96.5% (C sample). For the D sample, after a prior decrease after a time corresponding to 12 months of real aging, the peel adhesion increased almost to baseline for the maximum aging time. Verification of the peel adhesion is important knowledge in terms of the possibility of using adhesive tape to secure forensic traces. It also allows us to estimate the maximum functional shell-life of the designed product—adhesive tape to secure forensic traces.

**Figure 5:**
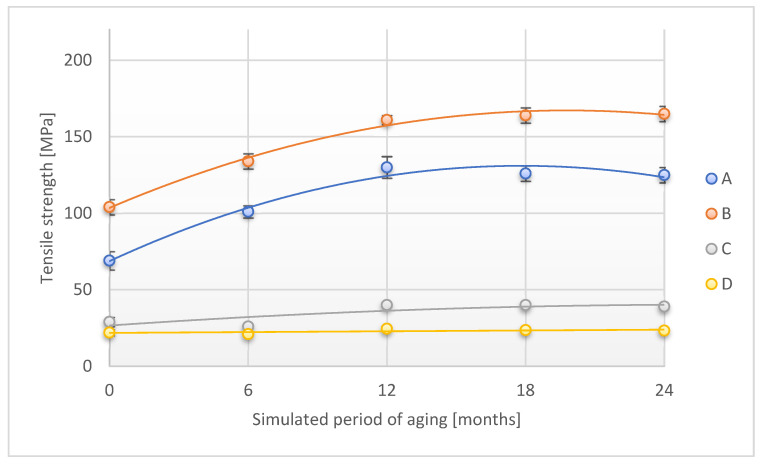
Change in tensile strength (direction 1) of the tested adhesion tapes during the accelerated aging using temperature: A—POLI TACK 850; B—POLI TACK 854; C—ORAGUARD 210; D—ORAGUARD 215.

The tensile strength tests, which are an important parameter characterizing the mechanical strength of the adhesive tape, were carried out in two directions: along (direction 1) and across (direction 2). The results of the study are shown in Figure 5.

In the case of tensile strength tests along the adhesive tape, a significant increase in the level of this parameter was observed during the first two periods of accelerated aging for A and B samples. Subsequently, the level of this parameter increased slightly, reaching the values of 125 ± 5 MPa and 165 ± 5 MPa, respectively, which is an increase of 81.2% and 58.6% compared to the initial value. The values of the parameter for the C sample increased disproportionately in relation to the above-discussed research material. The tensile strength verified during accelerated aging for the D sample did not significantly change its value.

**Figure 6:**
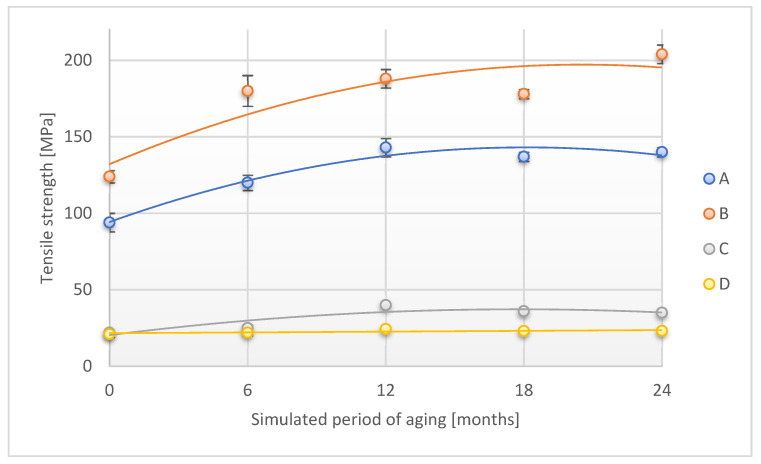
Change in tensile strength (direction 2) of the tested adhesion tapes during the accelerated aging using temperature: A—POLI TACK 850; B—POLI TACK 854; C—ORAGUARD 210; D—ORAGUARD 215.

A similar phenomenon was observed when testing the tensile strength parameter across the sample (Figure 6). In this case, the highest increase after 24 months of simulated aging was observed for sample B (increase by 64.5%), C (increase by 59.1%), and A (increase by 48.9%) in comparison to the initial value of this parameter. The D sample had an increase in tensile strength of 10.00% after a simulated period of 24 months.

**Figure 7:**
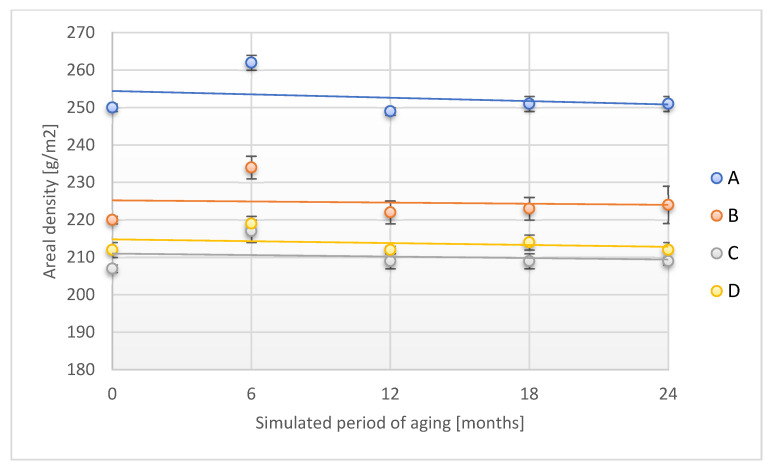
Change in areal density of the tested adhesion tapes during the accelerated aging using temperature and humidity: A—POLI TACK 850; B—POLI TACK 854; C—ORAGUARD 210; D—ORAGUARD 215.

The implementation of an additional aging factor—moisture—resulted in a slight increase in areal density for samples A, B, and C after a simulated storage time of 24 months (Figure 7).

In the case of the D sample, no change in areal density was observed after 24 months of simulated aging. It should be added that, after 6 months of simulated aging with the application of two aging factors temperature and humidity, a statistical increase in areal density was found for all tested samples of adhesive tapes from 3.3% to 6.4%, as compared to the initial value.

**Figure 8:**
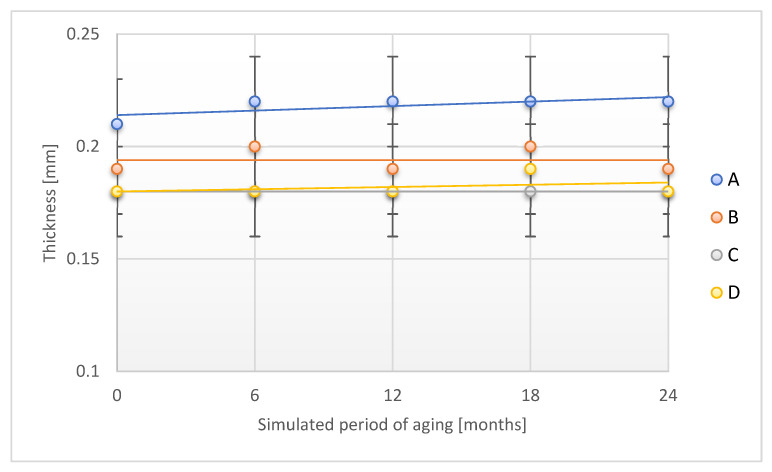
Change in thickness of the tested adhesion tapes during the accelerated aging using temperature and humidity: A—POLI TACK 850; B—POLI TACK 854; C—ORAGUARD 210; D—ORAGUARD 215.

The course of changes in the thickness of the tested samples of the adhesive tapes observed during the accelerated aging study using temperature and humidity is shown in Figure 8.

Studies have found no effect of the aging process with the use of temperature and humidity on B, C, and D sample thicknesses after a period of simulated 24 months of storage under real conditions. Compared to the original value, a slight increase in the thickness value for the A sample was found after the discussed aging period (an increase of 4.8%), which was consistent with the observations made for aging studies using only temperature as an aging factor.

**Figure 9:**
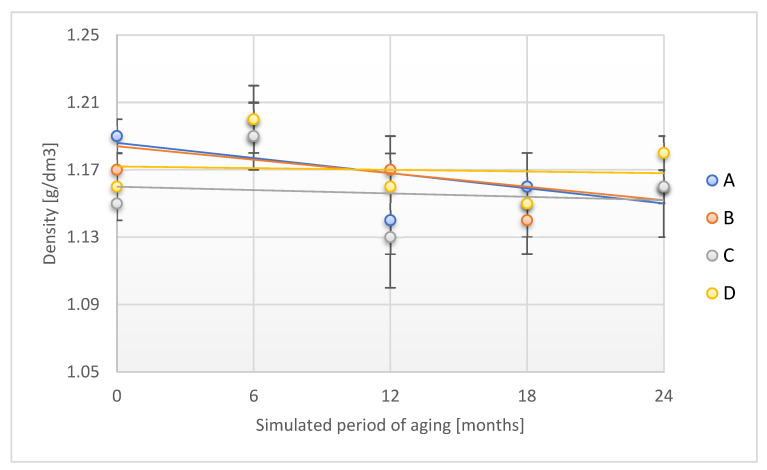
Change in density of the tested adhesion tapes during the accelerated aging using temperature and humidity: A—POLI TACK 850; B—POLI TACK 854; C—ORAGUARD 210; D—ORAGUARD 215.

The observed changes in the density of the tested adhesive tapes in terms of their use for securing forensic traces indicate a slight effect of an additional aging factor: humidity (Figure 9).

For A and B samples, a slight decrease in density was found after the simulated 24 months of aging (by 2.5% and 0.9%, respectively), whereas for C and D samples, a slight decrease in this parameter (by 0.9% and 1.7%, respectively) was observed. The trend of changes was similar to that of adhesion tape aging with a temperature factor, which confirms the stability of the discussed parameter during aging with the use of two aging factors: temperature and humidity.

It can be concluded that changes in the area of physical and mechanical properties during accelerated aging using both combinations of the aging factors are not significant from the point of view of the anticipated application of the tested tapes. The type of carrier material from which they are designed—PET and PVC—is important here, as it does not undergo significant changes in terms of the intended use.

**Figure 10:**
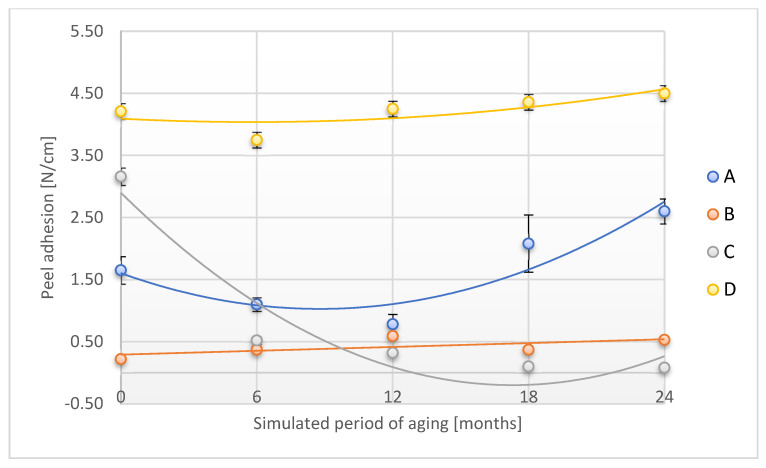
Change in peel adhesion of the tested adhesion tapes during the accelerated aging using temperature and humidity: A—POLI TACK 850; B—POLI TACK 854; C—ORAGUARD 210; D—ORAGUARD 215.

Figure 10 shows the changes observed for the peel adhesion of aged adhesive tapes using two factors: temperature and humidity.

In contrast to the case of aging with one factor, temperature, a significantly greater increase in this parameter was observed for A, B, and D samples after the simulated 24 months of aging (by: 57.6%, 141.9%, and 6.9%, respectively). The peel adhesion for the C tape decreased by 97.5% after this aging period. It should also be noted that after the first period of simulated aging of 6–12 months, a significant decrease in the value of peel adhesion was observed for samples A and C. Further, during the aging process, this parameter increased. The identified trends of changes indicate variations occurring during aging in the adhesive layer related to the reconstruction of the polymer layer structure, the variable impact of the aging process, and the applied aging factors on the important performance of adhesive tapes designed for use in the protection of forensic traces. Changes in adhesion tested in two combinations of aging agents are a direct result of changes in the polyacrylate layer, which is the adhesive layer of the tape. It seems that the aging process with the use of a temperature aging factor is more critical than in the case of using a combination of temperature and humidity, which may be related to the drying of the adhesive layer.

**Figure 11:**
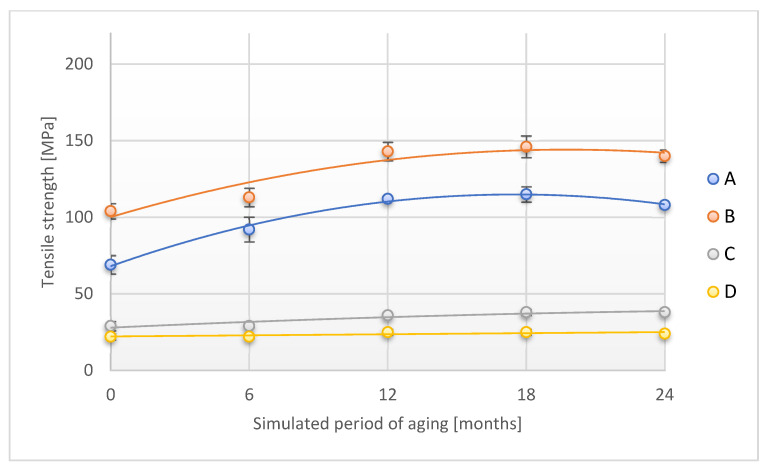
Change in tensile strength (direction 1) of the tested adhesion tapes during the accelerated aging using temperature and humidity: A—POLI TACK 850; B—POLI TACK 854; C—ORAGUARD 210; D—ORAGUARD 215.

**Figure 12:**
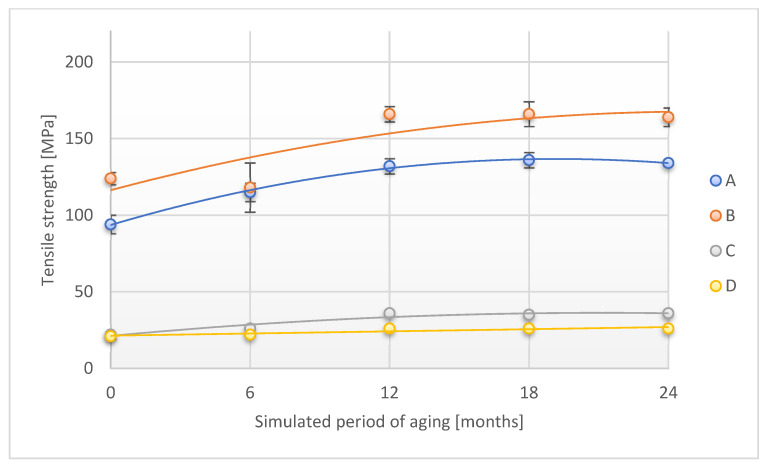
Change in tensile strength (direction 2) of the tested adhesion tapes during the accelerated aging using temperature and humidity: A—POLI TACK 850; B—POLI TACK 854; C—ORAGUARD 210; D—ORAGUARD 215.

Figure 11 and Figure 12 show the changes that occurred in the tensile strength of adhesive tapes during accelerated aging.

The course of the tensile strength dependence (tested in both directions) and the accelerated aging test is similar for the individual adhesive tape groups (A and B or C and D). The tensile strength increases significantly for tests A and B with the time of accelerated aging, except that the largest changes were observed at the test stage up to the simulated 12 months of storage, and at 24 months, a slight decrease in this parameter was observed compared to the value recorded for the 18th month of simulated storage. In the case of C and D samples, the increase in the value of the tested parameter was not as great as for the previously discussed samples. Stabilization of tensile strength values for tests C and D was also observed after simulated storage of 18 months.

The course of changes in the discussed parameter for both combinations of aging factors, temperature, and temperature and humidity, was similar and depended on the type of carrier material used—PET or PVC. The use of a temperature factor for the PET-originated tapes resulted in more significant increases in the parameter compared to aging when temperature and humidity were applied. The above phenomenon can be associated with a more significant impact of humidity on the PET degradation process, which is associated with changes in the polymer structure and, probably, with dynamic changes in its crystallinity. The above will be the subject of separate research.

Accelerated aging studies with UV exposure were performed by shortening the simulated duration of UV exposure, due to the significantly lower risk of exposure to UV radiation during storage under real conditions. The conducted research was also aimed at determining whether there is actually such a risk of changes in the physico-mechanical properties of the designed adhesive tapes for securing forensic traces during the UV exposure.

**Figure 13:**
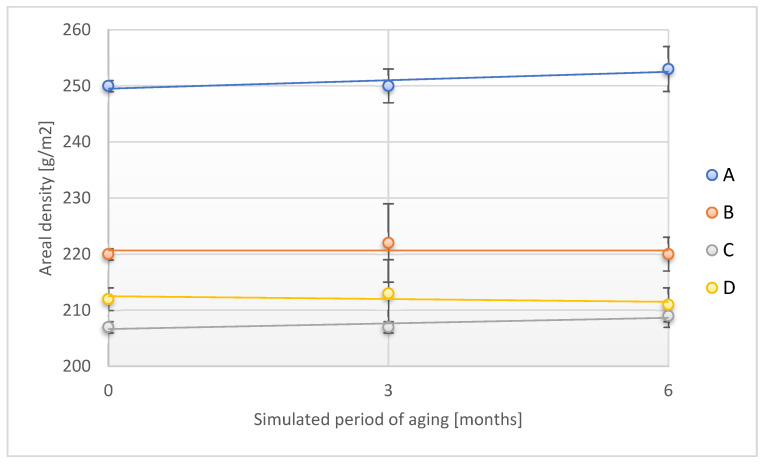
Change in areal density of the tested adhesion tapes during the accelerated aging using UV. A—POLI TACK 850; B—POLI TACK 854; C—ORAGUARD 210; D—ORAGUARD 215.

The course of changes in the areal density of the tested adhesive tapes under the influence of UV radiation is shown in Figure 13.

**Figure 14:**
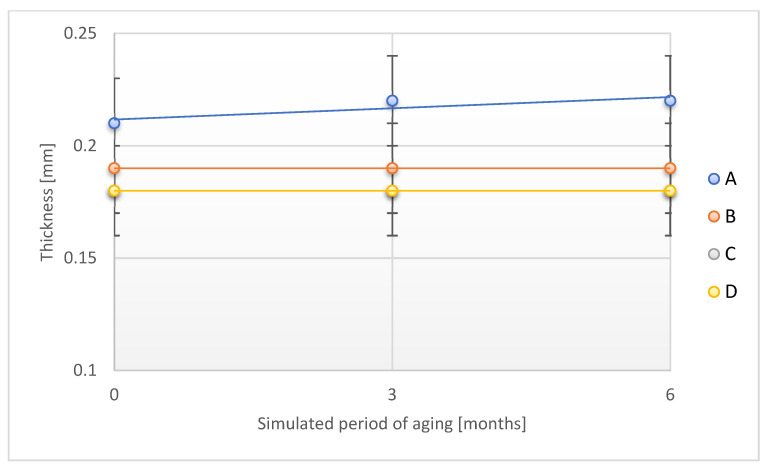
Change in thickness of the tested adhesion tapes during the accelerated aging using UV: A—POLI TACK 850; B—POLI TACK 854; C—ORAGUARD 210; D—ORAGUARD 215.

No statistically significant changes in the areal density of the tested samples were observed in the study. Therefore, it should be pointed out that the direct exposure of the designed adhesive tapes to UV radiation does not introduce a significant risk factor in terms of changes in the areal density of the tapes. Similar observations were performed for changes in the thickness of the adhesive tapes studied (Figure 14).

No statistical significance was found for the observed changes in the thickness of the tapes in individual stages of accelerated aging due to exposure to UV radiation. For samples C and D, exactly the same thickness values were obtained, which is why Figure 14 includes three curves.

**Figure 15:**
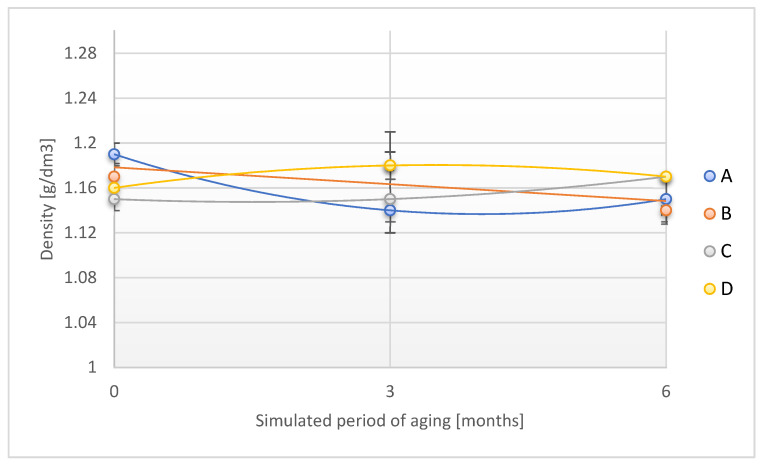
Change in density of the tested adhesion tapes during the accelerated aging using UV: A—POLI TACK 850; B—POLI TACK 854; C—ORAGUARD 210; D—ORAGUARD 215.

The course of the dependence of the density of the designed adhesive tapes on the time of accelerated aging by the UV factor is shown in Figure 15.

Similar to the areal density and thickness of the designed adhesive tapes, no significant changes were found in the value of the density in question in the individual stages of accelerated aging with the use of direct exposure of the sample to UV radiation. The above observations confirmed the slight effect of UV radiation on the physical properties of individual adhesive tapes.

**Figure 16:**
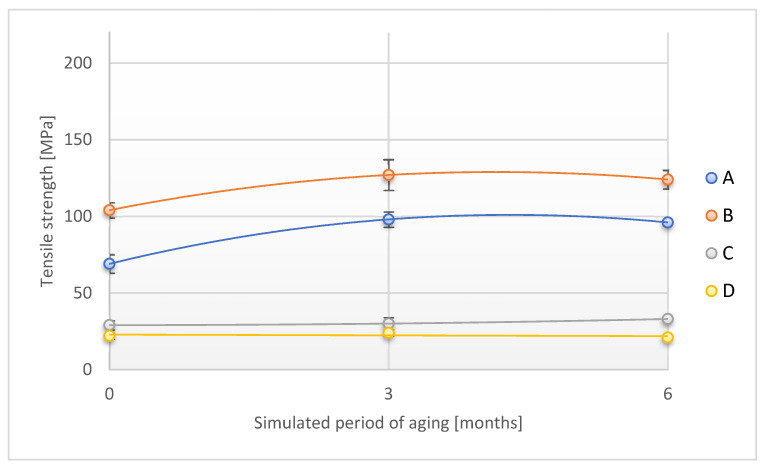
Change in tensile strength (direction 1) of the tested adhesion tapes during the accelerated aging using UV: A—POLI TACK 850; B—POLI TACK 854; C—ORAGUARD 210; D—ORAGUARD 215.

**Figure 17:**
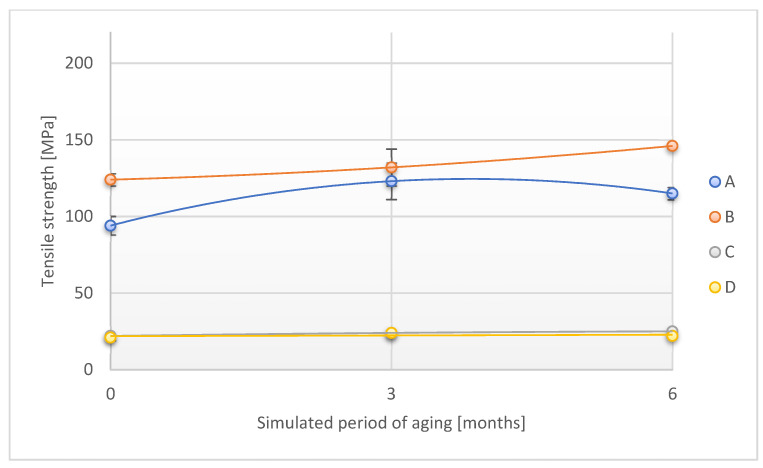
Change in tensile strength (direction 2) of the tested adhesion tapes during the accelerated aging using UV: A—POLI TACK 850; B—POLI TACK 854; C—ORAGUARD 210; D—ORAGUARD 215.

The relationship of tensile strength measured in two directions (lengthwise and across) on the time of exposure of adhesive tape samples to UV radiation is shown in Figure 16 and Figure 17.

The tensile strength values measured along the A and B tapes increased during the initial period of UV exposure (simulated period of 3 months under real conditions) and then decreased slightly. In the final period of UV exposure, the value of the parameter in question increased by 39.1% and 19.2%, respectively, as compared to the original value. A similar trend was observed for the C sample, while the value of the tested parameter slightly decreased for the D sample exposed for the simulated period of 6 months (by 4.6%).

When measuring mechanical strength across the B sample, an increase in this parameter was found throughout the UV exposure process (Figure 17). For samples A and C, a similar trend was observed when measuring tensile strength along the specimen. The D sample was characterized by a slight increase in the level of the discussed parameter (by 4.8%). Similar to the tested foils made of plastics for technical, medical, food, and office purposes, the tested films are more sensitive to thermal degradation than UV radiation, and changes in the tensile strength after exposure to UV radiation do not exceed 14% and after the thermal aging does not exceed 40% [35]. Tests of adhesive films used for securing criminal contact traces, taken directly from the protective packaging, were conducted based on previous research methodology on films made of PET, PA, and PP for technical, medical, food, and office applications. This study investigated the effect of accelerated aging simulation on the changes in output parameters of films made from PP, PET, and PA before and after specified time intervals. The environmental factors considered included temperature and UV radiation, focusing on the retention of physical and mechanical properties.

The selection of chemical reagents for chemical resistance tests of the designed adhesive tapes resulted from the analysis of risks related to the area of application of products for securing forensic traces and the factors that are used to secure, disclose, identify, and analyze forensic traces.

Table 2 presents the results of tests on the resistance of the designed adhesive tapes intended for securing forensic traces to chemical agents before and after the aging process with various aging agents.

Table 2 shows the chemical resistance of the designed adhesive tapes: initially and after the accelerated aging process against various aging agents. Chemical resistance was not assessed numerically, i.e., how long a sample exposed to a given chemical substance remains intact. The result was defined as positive if there were no changes on the tested surface and marked in Table 2 as “+”. In any other cases, the result was negative and marked in Table 2 as “-”.

A and B samples were resistant to all chemicals used for the verification tests prior to the accelerated aging process, while samples C and D were not resistant to a 32% solution of HCl, 65% solution of HNO_3_, and acetone.

The resistance against the tested chemicals for the A and B film samples was due to their chemical composition. Both samples were made using PET as a carrier, which is significantly more resistant to chemicals than PVC (film carrier for C and D samples).

The accelerated aging process carried out by various aging agents did not change the properties of the tested adhesive tapes intended for forensic protection (Table 2).

The accelerated aging process of the designed tapes did not adversely affect the change in resistance to the tested chemicals due to the negligible impact on the chemical structure of the tapes of the aging agents used. Among the factors used, even UV radiation did not cause such changes in the structure of the tapes that their resistance to the tested chemicals would change.

## 4. Conclusions

This article concerns the evaluation of the properties of tapes subjected to an accelerated aging process with the following factors: temperature 55 °C, or temperature 55 °C and humidity 55% or UV radiation (wavelength λ = 300–400 nm, irradiance 42 W/m^2^, temperature 35 °C (black bulb temperature 50 °C) and relative humidity 40%). Accelerated aging studies showed a noticeable effect on the mechanical properties of the designed adhesive tapes (mainly the film carrier) and the performance of the adhesive layer applied to the carrier. Changes in the tapes’ behavior were observed for all the aging factors used in the study; however, it seems that, after the analysis of the results, the most severe impact on the properties of adhesive tapes was the process of accelerated aging with the use of temperature alone. On the other hand, it should be stated that the exposure of the tapes to UV did not introduce significant changes to their physico-mechanical properties, taking into account the anticipated scope of potential application. However, this effect was caused by UV radiation on the functionality of the adhesive layer, which should be taken into account when defining possible restrictions related to use and storage. It should also be noted that the designed adhesive films, during storage, will be protected against UV radiation by specially designed direct and external packaging protecting the product against the risk of excessive exposure to UV radiation. The risk of exposure to UV radiation is carried by the very period of use of the product, i.e., the time between the securing of forensic evidence and its identification and secondary protection. This risk, taking into account the research results presented in the article, should be considered minimal and acceptable from the point of view of the assumed performance.

Chemical reagents selected due to the processes related to the protection, identification, analysis, and disclosure of forensic traces do not adversely affect only adhesive A and B tapes, which has also been confirmed in accelerated aging studies. This process carried out with various aging agents did not adversely affect the chemical resistance of the adhesive tapes assessed, which, in terms of the results of physicochemical tests, should predispose them to be used in the effective protection of forensic traces.

The next stage of the research will be to verify the impact of three factors of accelerated aging, temperature, humidity, and UV radiation, on the structural properties of tapes intended for the design of systems for securing criminal traces. The above will allow us to generate new knowledge in the area of changes occurring in the structure of carrier polymers (changes in the mechanical properties of the designed adhesive tapes under the influence of aging factors) and adhesive layers (changes in the strength of adhesion resulting from the action of aging factors).

The accumulation of tests will allow, as a result of a multi-criteria analysis [14,15,16], us to select the optimal raw material for the design of an advanced system for securing a criminal trace with confirmed stability of functional characteristics within the set time of product storage.

## Figures and Tables

**Table 1:** Characteristics of studied adhesion tapes used for the design of the tape for protection of the forensic traces.

Content	POLI TACK 850, (Sample A) polyacrylate-coated PET film, protected with siliconized PP film [Supplier: POLI-TAPE Klebefolien GmbH, Remagen, Germany]	POLI TACK 854, (Sample B) polyacrylate-coated PET film, protected with siliconized PP film [Supplier: POLI-TAPE Klebefolien GmbH, Remagen Germany]	ORAGUARD 210, (Sample C) PVC foil, UV protected, polyacrylate coated on one side, silicone paper protected [Supplier: ORAFOL, Oranienburg, Germany]	ORAGUARD 215, (Sample D) PVC foil, UV protected, coated on one side with polyacrylate solvent, protected with silicone paper [Supplier: ORAFOL, Oranienburg, Germany]
Carrier	Clear matte polyester film	Clear matte polyester film	PVC foil	PVC foil
Adhesion layer	Clear acrylic lacquer	Clear acrylic lacquer	Solvent-based polyacrylate, permanent, transparent	Solvent-based polyacrylate, permanent, transparent

**Table 2:** Chemical resistance of the designed adhesive tapes before and after the aging process to various aging agents.

Sample	Simulated Aging Time [Month]	Chemicals														
		40% NaOH			32% HCl			65% HNO_3_			Gasoline			Acetone		
		T ^(1)^	T+W ^(2)^	UV ^(3)^	T ^(1)^	T+W ^(2)^	UV ^(3)^	T ^(1)^	T+W ^(2)^	UV ^(3)^	T ^(1)^	T+W ^(2)^	UV ^(3)^	T ^(1)^	T+W ^(2)^	UV ^(3)^
A (POLI TACK 850)	0	+	+	+	+	+	+	+	+	+	+	+	+	+	+	+
	3	n/a	n/a	+	n/a	n/a	+	n/a	n/a	+	n/a	n/a	+	n/a	n/a	+
	6	+	+	+	+	+	+	+	+	+	+	+	+	+	+	+
	12	+	+	n/a	+	+	n/a	+	+	n/a	+	+	n/a	+	+	n/a
	18	+	+	n/a	+	+	n/a	+	+	n/a	+	+	n/a	+	+	n/a
	24	+	+	n/a	+	+	n/a	+	+	n/a	+	+	n/a	+	+	n/a
B (POLI TACK 854)	0	+	+	+	+	+	+	+	+	+	+	+	+	+	+	+
	3	n/a	n/a	+	n/a	n/a	+	n/a	n/a	+	n/a	n/a	+	n/a	n/a	+
	6	+	+	+	+	+	+	+	+	+	+	+	+	+	+	+
	12	+	+	n/a	+	+	n/a	+	+	n/a	+	+	n/a	+	+	n/a
	18	+	+	n/a	+	+	n/a	+	+	n/a	+	+	n/a	+	+	n/a
	24	+	+	n/a	+	+	n/a	+	+	n/a	+	+	n/a	+	+	n/a
C (ORAGUARD 210)	0	+	+	+	-	-	-	-	-	-	+	+	+	-	-	-
	3	n/a	n/a	+	n/a	n/a	-	n/a	n/a	-	n/a	n/a	+	n/a	n/a	-
	6	+	+	+	-	-	-	-	-	-	+	+	+	-	-	-
	12	+	+	n/a	-	-	n/a	-	-	n/a	+	+	n/a	-	-	n/a
	18	+	+	n/a	-	-	n/a	-	-	n/a	+	+	n/a	-	-	n/a
	24	+	+	n/a	-	-	n/a	-	-	n/a	+	+	n/a	-	-	n/a
D (ORAGUARD 215)	0	+	+	+	-	-	-	-	-	-	+	+	+	-	-	-
	3	n/a	n/a	+	n/a	n/a	-	n/a	n/a	-	n/a	n/a	+	n/a	n/a	-
	6	+	+	+	-	-	-	-	-	-	+	+	+	-	-	-
	12	+	+	n/a	-	-	n/a	-	-	n/a	+	+	n/a	-	-	n/a
	18	+	+	n/a	-	-	n/a	-	-	n/a	+	+	n/a	-	-	n/a
	24	+	+	n/a	-	-	n/a	-	-	n/a	+	+	n/a	-	-	n/a

(1) accelerated aging with temperature; (2) accelerated aging with temperature and humidity; (3) accelerated aging with UV; “+”—resistant; “-”—non-resistant; “n/a”—not applicable.

## Data Availability

The raw data supporting the conclusions of this article will be made available by the authors on request due to privacy/ethics.
